# Extraction and Hypolipidemic Activity of Low Molecular Weight Polysaccharides Isolated from *Rosa Laevigata* Fruits

**DOI:** 10.1155/2020/2043785

**Published:** 2020-10-22

**Authors:** Xuejiao Zhang, Yihong Hu, Chenzhong Jin, Weiguo Wu

**Affiliations:** ^1^College of Food Science and Technology, Hunan Agricultural University, Changsha, 410128 Hunan, China; ^2^Collaborative Innovation Center for Field Weeds Control, Hunan University of Humanities, Science and Technology, Loudi, 417000 Hunan, China

## Abstract

Three novel low molecular weight polysaccharides (RLP-1a, RLP-2a, and RLP-3a) with 9004, 8761, and 7571 Da were first obtained by purifying the crude polysaccharides from the fruits of a traditional Chinese medicinal herb *Rosae Laevigatae*. The conditions for polysaccharides from the *R. Laevigatae* fruit (RLP) extraction were optimized by the response surface methodology, and the optimal conditions were as follows: extraction temperature, 93°C; extraction time, 2.8 h; water to raw material ratio, 22; extraction frequency, 3. Structural characterization showed that RLP-1a consisted of rhamnose, arabinose, xylose, glucose, and galactose with the ratio of 3.14 : 8.21 : 1 : 1.37 : 4.90, whereas RLP-2a was composed of rhamnose, mannose, glucose, and galactose with the ratio of 1.70 : 1 : 93.59 : 2.73, and RLP-3a was composed of rhamnose, arabinose, xylose, mannose, glucose, and galactose with the ratio of 6.04 : 26.51 : 2.05 : 1 : 3.17 : 31.77. The NMR analyses revealed that RLP-1a, RLP-2a, and RLP-3a contained 6, 4, and 6 types of glycosidic linkages, respectively. RLP-1a and RLP-3a exhibited distinct antioxidant abilities on the superoxide anions, 1,1-diphenyl-2-picrylhydrazyl (DPPH), and hydroxyl radicals *in vitro*. RLPs could decrease the serum lipid levels, elevate the serum high-density lipoprotein cholesterol levels, enhance the antioxidant enzymes levels, and upregulate of FADS2, ACOX3, and SCD-1 which involved in the lipid metabolic processes and oxidative stress in the high-fat diet-induced rats. These results suggested that RLPs ameliorated the high-fat diet- (HFD-) induced lipid metabolism disturbance in the rat liver through the peroxisome proliferator-activated receptor (PPAR) signaling pathway. Low molecular weight polysaccharides of RLP could be served as a novel potential functional food for improving hyperlipidemia and liver oxidative stress responses.

## 1. Introduction

*Rosae Laevigatae* is a perennial shrub belonging to the Rosaceae family, and its fruits have been used widely as edible food and medicinal herb in the tropical and subtropical areas of Asia for thousands of years. The fruits are rich in vitamin C, polysaccharides, triterpenoid acids, steroids, polyphenols, saponins, and flavonoids [1, 2]. In China, they are used as the foodstuff in the traditional brewing of wine, for the extraction of brown pigments, and the preparation of the healthy vinegar-based beverages [3]. Previous studies also indicated that this medicinal plant had the effects on antioxidation [4], antiapoptosis [5], reducing inflammation [2], inhibiting arterial sclerosis [1], and hepatoprotective activity [6]. Polysaccharides, flavonoids, and saponins from these fruits have been confirmed as primary active constituents by previous pharmacological researches [7, 8]. Among them, polysaccharides are the main components with approximately 260.5 mg/g in total and are of great importance to humans [1], but few documents on the extraction, characterization, and biological activities of the polysaccharides from *R. laevigata* fruits (RLP) have been published.

Plant polysaccharides widely exist in plant organs including flowers, roots, leaves, and fruits. In recent years, plant polysaccharides have been attracting great attention due to their natural and nontoxic properties with no side effects. Their bioactivities and pharmacological functions were identified in terms of antioxidation [9], anticancer [10], anti-inflammatory [11], antimicrobial [12], hepatoprotective [13], hypolipidemic, and hypoglycemic activities [14, 15], indicating a wide application prospect in medicine. However, early studies have shown that the biological activity of polysaccharide was closely related to its chemical structure, molecular weight, and monosaccharide composition [16]. High molecular weight of polysaccharides limits their applications owing to their physical properties including low solubility and complex structures [17]. The molecular weight of polysaccharides over 10 kDa in weight had poor solubility, was not conducive to the digestion and absorption *in vivo*, and subdued the biological activities, whereas the low molecular weight polysaccharides less than 10 kDa in weight had relatively higher activities and were of greater significance to human health [18–20]. Polysaccharides with larger molecular weight appeared high apparent viscosity, poor water solubility, and complex structures and conformations [20]. They are difficult to enter into cells or attach to the receptors to exert their biological effects [21]. The clinical application of natural polysaccharides has been restricted due to their large molecular weights and low solubility. The separation of polysaccharides with low molecular weight is becoming a hotspot.

At present, there are no reports on the separation of polysaccharides with low molecular weight less than 10 kDa from the *R. Laevigatae* fruits (RLPs). The main researches focused on the hypolipidemic, antioxidant, and immunomodulatory activities of high molecular weight polysaccharides of *R. Laevigatae* fruits [1, 2]. It is also unclear whether RLPs have better antioxidant or hypolipidemic activities. Thus, a detailed structural, antioxidant, and hypolipidemic analyses of RLPs are necessary to support their potential application for functional food ingredients.

In this study, three low molecular weight RLPs, namely, RLP-1a, RLP-2a, and RLP-3a, were first isolated from *R. Laevigatae* fruits by fractionation and purification. The primary chemical structures of RLP-1a, RLP-2a, and RLP-3a were characterized by the Fourier transform infrared (FT-IR), gas chromatography-mass spectrometry (GC-MS), and nuclear magnetic resonance (NMR) analyses. The antioxidant and hypolipidemic activities of RLPs composed of RLP-1a, RLP-2a, and RLP-3a were evaluated by investigating their effects on lipid profiles, antioxidant enzyme activities, and lipid metabolic gene expressions in high-fat diet- (HFD-) induced rat. The study could provide implications for the utilization of low molecular weight RLPs in functional food to improve blood lipid metabolism.

## 2. Materials and Methods

### 2.1. Materials and Chemicals

The *R. Laevigatae* fruits were purchased from a local market in Hunan, China. L-ascorbic acid and 1,1-diphenyl-2-picrylhydrazyl (DPPH) were purchased from Sigma-Aldrich (St. Louis, Missouri, USA). Diethylaminoethyl- (DEAE-) cellulose and Sepharose CL-4B were purchased from Whatman (Sanford, ME, UK). All other chemicals used were of analytical-reagent grade.

### 2.2. Extraction of Polysaccharides from *R. Laevigatae* Fruits

After dried, crushed, and screened for 60 meshes, the *R. Laevigatae* fruit powders were obtained. The powders were defatted with petroleum ether (boiling point 60-90°C) and then pretreated with 90% ethanol (v/v) for several times to remove pigments, monosaccharides, and phenolic compounds until the solution was colorless. After centrifuged at 4000 × g for 20 min, the sediment was vacuum-dried to constant weight. Each dried and pretreated sample (10 g) was extracted with deionized water at the designated extraction conditions. The conditions were controlled by varying temperature, extraction time, water to raw material ratio, and extraction frequency. After centrifugation at 4000 × g for 20 min, the supernatant was dialyzed for 48 h against deionized water to remove oligosaccharides. The liquid obtained in a dialysis bag was precipitated by overnight incubation against ethanol to a final concentration of 80% (v/v). The precipitate was collected by centrifugation at 4000 × g for 15 min and deproteinated by Sevag method for five times [1, 22]. After lyophilized, the polysaccharide content was measured using the phenol-sulfuric acid method with D-glucose as standard [23].

### 2.3. Response Surface Methodology

Four variables (*X*_1_, extraction temperature; *X*_2_, extraction time; *X*_3_, water to raw material ratio; and *X*_4_, extraction frequency) at five levels (*X*_1_ with 80, 85, 90, 95, and 100°C; *X*_2_ with 1, 1.5, 2, 2.5, and 3 h; *X*_3_ with 1 : 16, 1 : 18, 1 : 20, 1 : 22, and 1 : 24; and *X*_4_ with 1, 2, 3, 4, and 5) were performed according to the experiment results of single factor effect on polysaccharide extraction. Thirty independent variable combinations were designed by central composite design (CCD), each of which was performed in triplicate in a randomized order. The response value of each trial was calculated as the average of the duplicates. The CCD data were analyzed by the multiple regression.

### 2.4. Isolation and Purification

About 100 g dried powder of *R. Laevigatae* fruits was used as raw material. After defatted and pretreated, the polysaccharides were extracted with 2 L of distilled water at 90°C for 2 h. Deproteinated by Sevag method and dialyzed to intercept molecular weight greater than 3500 Da, the liquid obtained in the dialysis bag was precipitated overnight against ethanol to a final concentration of 30% (v/v). After centrifugation at 4000 × g for 20 min, the supernatant was collected and further precipitated overnight to a final concentration of 50% (v/v). The supernatant was treated as above with ethanol to a final concentration of 80% (v/v). After centrifuged at 4000 × g for 20 min and freeze-dried, the crude RLP was obtained.

Approximately 30 mg crude RLP in 10 mL water was loaded onto a diethylaminoethyl- (DEAE-) cellulose column (2.6 × 80 cm) preequilibrated with water and eluted in NaCl gradient solution (0-1.5 M) at the flow rate of 1 mL/min. Each elution tube was collected and monitored for carbohydrate content as abovementioned. Finally, the tubes of each carbohydrate-positive fraction were pooled together separately, then dialyzed and lyophilized. The products were further purified by gel filtration chromatography on a Sepharose CL-4B column (2.6 × 80 cm) with water at a flow rate of 1 mL/min, and the different fractions were pooled together separately, dialyzed, and lyophilized, and then the purified RLPs were obtained.

### 2.5. RLP Analysis

#### 2.5.1. Molecular Weight Determination

The homogeneity and molecular weight of the purified RLPs were determined by high performance gel permeation chromatography (HPGPC), which was performed on a HPLC-1525 liquid chromatography instrument (Waters, Massachusetts, USA) with a TSK-GEL G5000 PWXL column (7.8 × 300 mm) series connected with a TSK-GEL G3000 PWXL column (7.8 × 300 mm) (Tosoh, Tokyo, Japan). The column was eluted with 0.02 M Na_2_SO_4_ with a flow rate of 0.6 mL/min and detected by a RID-2410 detector. The sample (2 mg) was dissolved in the mobile phase and centrifuged, and the volume of 20 *μ*l sample was injected in each run. The molecular weight was estimated by reference to the calibration curve made from a standard dextran solution consisting T-400, T-300, T-200, T-100, T-40, T-20, T-10, and T-5 [24].

#### 2.5.2. Monosaccharide Composition Analysis

Monosaccharide compositions of the purified RLPs were estimated through gas chromatography-mass spectrometry (GC-MS) 6890-5975 (Agilent Co., Colorado Springs, CO, USA). The sample (10 mg) was hydrolyzed in 2 mL of 4 M trifluoroacetic acid (TFA) for 3 h at 120°C in a sealed glass tube. The hydrolysate was evaporated to dry under N_2_ and dissolved in 1 mL pyridine with 10 mg hydroxylamine at 90°C for 30 min. Afterward, 1 mL acetic anhydride was added to the solution and allowed to react at 90°C for 30 min before being filtered through 0.22 *μ*m syringe filters (Whatman, Sanford, ME, UK). The final product was analyzed by GC-MS. As external standards, the following monosaccharides were converted to their acetylated derivatives and analyzed: rhamnose, ribose, fucose, arabinose, xylose, mannose, glucose, galactose, and fructose.

#### 2.5.3. Ultraviolet and Fourier Transformation Infrared Spectrum Analysis

Ultraviolet spectra of the purified RLPs were obtained on a T60 UV-Vis spectrophotometer (Shimadzu, Kyoto, Japan). The scanning range was 190-600 nm at 5 nm interval, resulting in 82 points spectra for each sample.

Fourier transformation infrared spectra (FT-IR) for the samples were recorded on a Nicolet 6700 IR spectrometer (Thermo Fisher Scientific, Waltham, MA, USA). The dried samples were ground with spectroscopic grade KBr powder for FT-IR measurement in the wavenumber range of 4000-400 cm^−1^ [24].

#### 2.5.4. Nuclear Magnetic Resonance Spectroscopy

Purified RLP (60 mg) was dissolved in 0.5 mL D_2_O for the nuclear magnetic resonance spectroscopy (NMR) analysis. Spectra were recorded by a Bruker DRX-600 spectrometer (Rheinstetten, Germany) at 25°C. The one-dimensional (^1^H and ^13^C) NMR was performed using the standard Bruker software TopSpin. ^1^H chemical shifts were referenced to residual D_2_O at 4.71 ppm at 25°C as the internal standard, whereas ^13^C chemical shifts were determined with acetone (*δ*^1^H 2.19 ppm and ^13^C 31.50 ppm) that was calibrated externally, and the chemical shift units were expressed as ppm [25].

#### 2.5.5. Morphological Analysis

The morphology of the purified RLPs was analyzed by an environment scanning electron microscope (JSM-7500F, JEOL, Japan). The dried powder of samples was placed on tapes, sputtered with gold, and determined with an acceleration voltage of 5 kV.

### 2.6. *In Vitro* Antioxidant Activity Assays

#### 2.6.1. Hydroxyl Radical Scavenging Assay

The scavenging activity of samples against hydroxyl radicals was measured as described [26] with some modifications. Various samples (1.0 mL each) at the concentrations of 0.2, 0.4, 0.6, 0.8, 1.0, and 0.2 mg/mL were mixed with 1 mL of 1.5 mM ethylenediaminetetraacetic acid-Fe(II), 1 mL of 10 mM H_2_O_2_, and 1 mL of 9 mM salicylic acid. After incubation at 37°C for 60 min, the absorbance of the solution was measured at 560 nm with ascorbic acid as the positive control. The scavenging activity of the sample toward hydroxyl radicals was calculated using the following formula:

(1) $$\begin{array}{c} \text{Scavenging rate }\left(\%\right)=\left(1-\frac{A_{i}}{A_{0}}\right)\times 100, \end{array}$$

where *A*_0_ was the blank absorbance, and *A*_*i*_ was the absorbance of sample or ascorbic acid.

#### 2.6.2. DPPH Scavenging Assay

The scavenging activity of samples against DPPH radicals was determined according to the method [26] with modifications. The reaction mixture comprised 1 mL DPPH (0.1 mM in ethanol) and 1 mL sample solution (0.2, 0.4, 0.6, 0.8, 1.0, and 1.2 mg/mL). The mixture was incubated at 25°C for 30 min in dark, and the absorbance of the mixture was determined at 517 nm, and ascorbic acid was used as the positive control, and 95% ethanol was the blank control. The DPPH radical scavenging ability was calculated using formula (1).

#### 2.6.3. Superoxide Radical Scavenging Assay

The scavenging activity for superoxide anions radical was performed with the methods [9, 27] with modifications. About 4.5 mL 50 mM Tris–HCl buffer (pH 8.2) was mixed with 4.2 mL of deionized water. After incubation at 25°C for 20 min, 1 mL sample solution and 0.4 mL pyrogallic acid were added to the mixture. The mixture was rapidly shaken and incubated at 25°C for 5 min. Subsequently, 8 mM HCl was added to the mixture to terminate the reaction. The absorbance of the mixture was measured at 320 nm with ascorbic acid as the positive control. The scavenging ability of the sample toward superoxide radicals was calculated using formula (1).

### 2.7. Design of Animal Experiments

Normal chow and purified high-fat diet (45% fat) were purchased from MediScience Ltd. (Yangzhou, China). Eight-week-old male rats were provided by Hunan SJA Laboratory Animal Co., Ltd. They were acclimatized in the controlled environment (temperature 22 ± 2°C; 60 ± 10% humidity; and a 12 h/12 h light/dark cycle) with free access to water and standard chow. All rats were fed for one week and designated into four groups, with ten rats on each group, namely, the control group (CN), the high-fat diet group (HFD), the high-dose RLP group (HRLP), and the low-dose RLP group (LRLP). The CN group was fed with normal chow and treated with 5% acacia gum solution. The HFD group was fed with HFD and treated with 5% acacia gum solution. The HRLP group and LRLP group were fed with HFD + 800 mg/kg RLP and HFD + 200 mg/kg RLP, respectively. After 12 weeks, the rats were sacrificed by cervical dislocation after anesthesia. The blood samples were centrifuged at 7000 × g for 10 min at 4°C. Serum was immediately collected, frozen, and stored at -80°C until analysis [28]. Tissues were snap frozen by liquid nitrogen and preserved in the -80°C refrigerator for further analysis.

### 2.8. Biochemical Determination of Serum and Liver

The serum total cholesterol (TC), triglycerides (TG), high-density lipoprotein (HDL-C), low-density lipoprotein (LDL-C), the weak absorption peaks at 2935, and free fatty acid (FFA) concentrations were tested by an automatic biochemical analyzer (Beckman Coulter, Atlanta, Georgia, USA). The levels of alanine transaminase (ALT), aspartate transaminase (AST), and glutamyl transpeptidase (GGT) in serum and the contents of catalase (CAT), superoxide dismutase (SOD), and glutathione oxidase (GSH-Px) in liver supernatant were tested following instructions of the kits (Beckman Coulter, Beverly, USA).

### 2.9. Transcriptome Analysis

Total RNA of liver tissue was extracted by Trizol method. RNA integrity was assessed using the RNA Nano 6000 Assay Kit of the Bioanalyzer 2100 system (Agilent Technologies, CA, USA). PCR was performed with Phusion High-Fidelity DNA polymerase, Universal PCR primers, and Index (X) Primer. The PCR products were purified by AMPure XP system (Beckman Coulter, Beverly, USA), and the library quality was assessed on the Agilent Bioanalyzer 2100 system.

Differential expression analyses of two conditions/groups (two biological replicates per condition) were performed using the DESeq2 R package. The resulting *P* values were adjusted using the Benjamini and Hochberg's approach for controlling the false discovery rate. Genes with an adjusted *P* value < 0.05 found by DESeq2 were assigned as differentially expressed. Corrected *P* value < 0.05 and absolute fold change > 2 were set as the threshold for significantly differential expression.

Gene ontology (GO) enrichment analysis of differentially expressed genes was implemented by the cluster Profiler R package, in which gene length bias was corrected. GO terms with corrected P-value less than 0.05 were considered significantly enriched by differential expressed genes. We used cluster Profiler R package to test the statistical enrichment of differential expression genes in KEGG pathways. Differentially expressed genes identified by transcriptome analysis were validated using qRT-PCR.

### 2.10. Statistical Analysis

Design Expert 8.0.6 was used to design the response surface experiment. Unless otherwise indicated, the results were expressed as mean ± SD (standard deviation). SPSS 17.0 (SPSS Inc., Chicago, IL, USA) was used to analyze the experimental data by one-way *ANOVA*. Confidence levels for statistical significance were set at *P* < 0.05.

## 3. Results and Discussion

### 3.1. Optimization of the Extraction Conditions for Polysaccharides from *R. Laevigatae* Fruit

The design matrix and corresponding results obtained from CCD for determining the effects of four independent variables *X*_1_, *X*_2_, *X*_3_, and *X*_4_, respectively, were listed in Table 1. The results showed that the extraction rate ranged from 7.19% to 9.49%. The multiple regression analysis performed on the experimental data revealed that the relationship between the response and test variables was described by the following second-order polynomial equation:

(2) $$\begin{array}{c} Y\left(\%\right)=-55.329+0.912X_{1}-1.974X_{2}+1.553X_{3}+5.600X_{4}-0.016X_{1}X_{2}+0.023X_{1}X_{3}+0.004X_{1}X_{4}+0.288X_{2}X_{3}-0.288X_{2}X_{4}-0.147X_{3}X_{4}-0.007{X_{1}}^{2}-0.284{X_{2}}^{2}-0.092{X_{3}}^{2}-0.399{X_{4}}^{2}, \end{array}$$

where *Y*: the extraction yield (%); *X*_1_: extraction temperature (°C); *X*_2_: extraction time (h); *X*_3_: water to raw material ration (mL/g); *X*_4_: extraction frequency.

The fitting statistics of the extraction rate *Y* to the selected quadratic model were shown in Table 2. *ANOVA* of the quadratic regression model showed that the determination coefficient *R*^2^ was 0.9128, indicating that only 8.72% of the total variation could not be explained by the model. The adjusted determination coefficient (*R*_adj_^2^ = 0.8315) also confirmed that the model was highly significant. The model was thus adequate for prediction within the range of experimental variables. The regression coefficient values were listed in the table. *P* values were calculated to determine the significance of various coefficients and described patterns of interaction between variables. A smaller *P* value indicated a more significant corresponding coefficient. Statistical analysis showed that extraction time (*X*_2_) was the most important independent variable that affected the extraction rate, followed by the extraction temperature (*X*_1_), the extraction frequency (*X*_4_), and the water to raw material ratio (*X*_3_).

**Figure 1:**
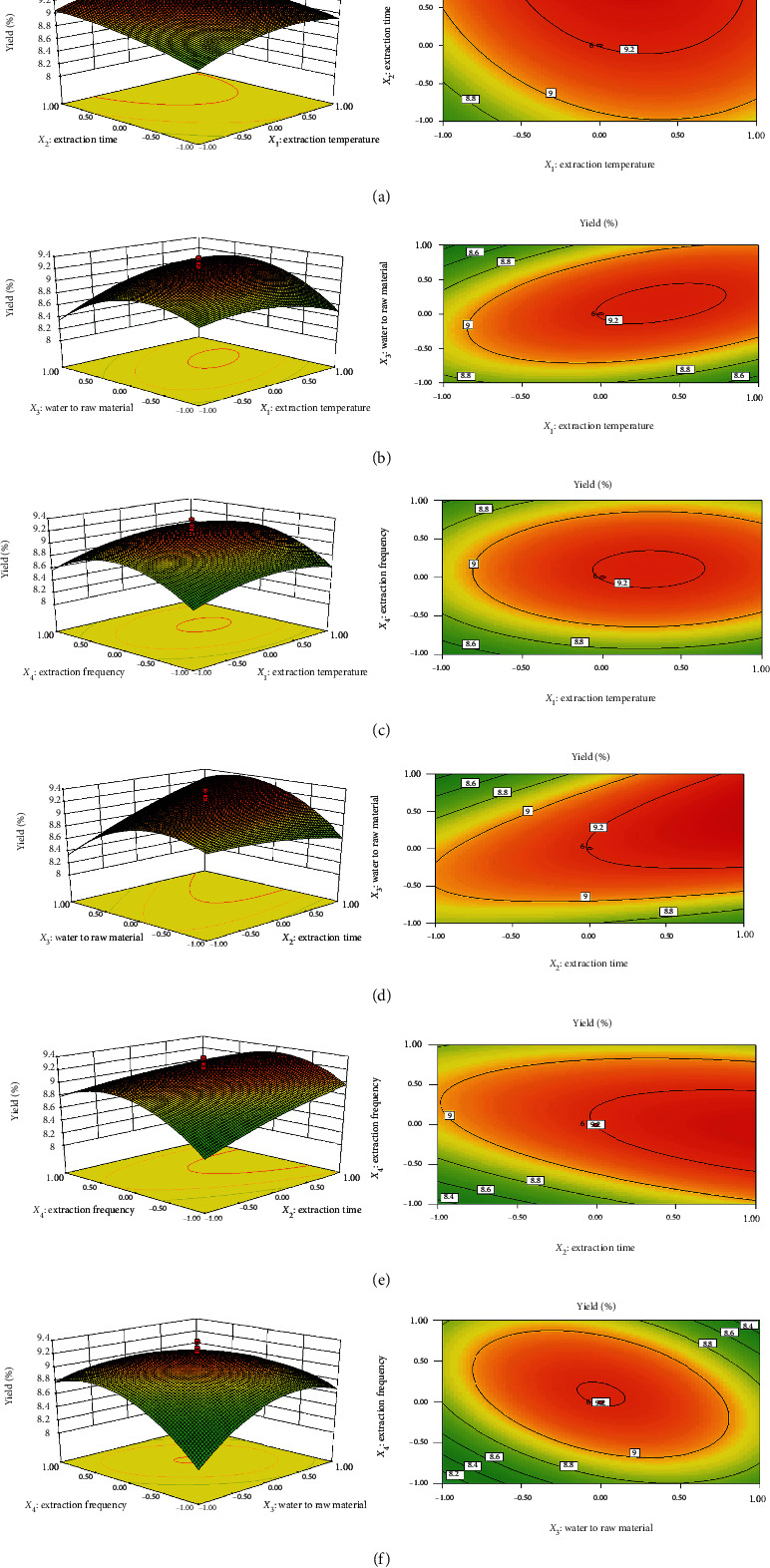
The response surface plots and contour plots of the interaction effects of four different variables (*X*_1_: extraction temperature, °C; *X*_2_: extraction time, h; *X*_3_: ratio of water to raw material, mL/g; *X*_4_: extraction frequency) on the response yield of RLP.

The 3D response surface and contour plots of the CCD were obtained using the Design Expert Software (version 8.0.6). The effects and interactions of the four factors in response surface were directly reflected by the steepness of the trend of response surface curve and the density of contour plots. As shown in Figure 1(a), the response surface of extraction time was relatively steep and the contour lines were relatively dense. However, the response surface of extraction temperature was relatively smooth, and the contour lines were relatively sparse. Effect of extraction time on the extraction yield of RLP was more significant than that of the extraction temperature. The interaction between extraction temperature and water to raw material ratio on the extraction yield was shown in Figure 1(b). Similarly, the extraction yield did not change obviously by increasing the extraction temperature and water to raw material ratio. As shown in Figure 1(c), the extraction yield did not change significantly with the change of extraction temperature and extraction frequency. Response surface of extraction temperature and extraction frequency was relatively smooth, and the contour was relatively sparse. It was worth noting that the response surface of extraction time was relatively steep, and the contour lines were relatively dense, compared with those of water to raw material ratio and extraction frequency (Figures 1(d) and 1(e)), suggesting that extraction time was the main factor for the extraction yield of RLP.

According to the analysis by the Design Expert Software, the optimal extraction conditions were as follows: extraction temperature, 93°C; extraction time, 2.8 h; water to raw material ratio, 22; and extraction frequency, 3. Among the four extraction parameters, the extraction time was the most significant factor affecting polysaccharides yield; the extraction temperature, extraction frequency, and water to raw material ratio were not significant factors affecting yield within the test range used. The suitability of the model equation for predicting optimum response values was determined using the optimized condition to validate and predict the values of the responses using the model equation. Compared with the predicted value 9.56, a mean value of 9.61 ± 0.08 (*n* = 3) was obtained from the experiments. This result validated the adequacy of the RSM model for describing the extraction process.

### 3.2. Isolation and Purification

**Figure 2:**
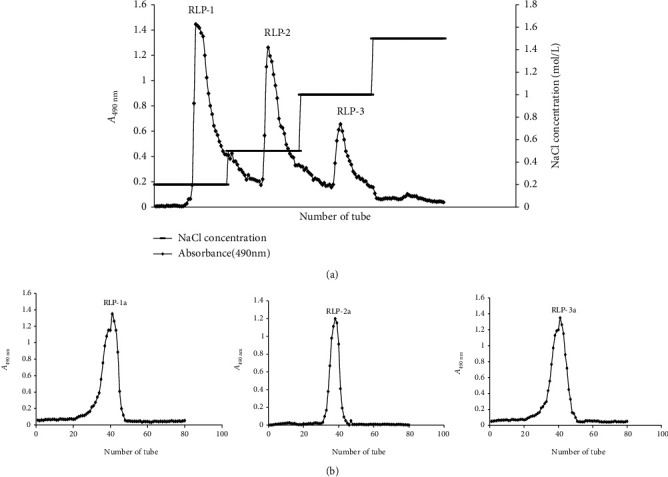
Elution curve of RLP on DEAE-cellulose DE-52 column (a) and elution curve of RLP-1, RLP-2, and RLP-3 on Sepharose CL-4B column (b).

The crude RLP was obtained and loaded onto a DEAE-cellulose column equilibrated with a linear NaCl gradient. Three independent elution peaks, RLP-1, RLP-2, and RLP-3 (Figure 2(a)) were detected by the phenol-sulfuric acid method. The three fractions were collected for the subsequent purification with Sepharose CL-4B column and presented single peaks RLP-1a, RLP-2a, and RLP-3a, respectively (Figure 2(b)). Then, RLP-1a, RLP-2a, and RLP-3a were collected for further structural characterization and antioxidant activity assays.

### 3.3. Molecular Weight of RLPs

**Figure 3:**
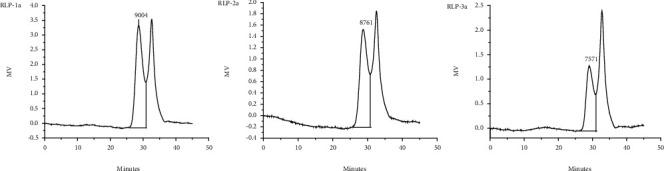
HPGPC chromatogram of low molecular weight soluble polysaccharides RLP-1a, RLP-2a, and RLP-3a from *R. Laevigatae* fruits.

Based on the distribution of molecule weight as determined by HPGPC analysis, RLP-1a, RLP-2a, and RLP-3a fractions produced a single symmetrical and narrow peak (Figure 3), indicating they were homogeneous polysaccharides. According to the calibration curve, the average molecular weights of RLP-1a, RLP-2a, and RLP-3a were estimated to be about 9004 Da, 8761 Da, and 7571 Da, respectively. In previous studies, Yu et al. [1] obtained two major fractions which were 21.5 kDa and 16.1 kDa. Zhan et al. [2] obtained an acid polysaccharide with the Mw of 137.1 kDa from *R. Laevigatae* fruits. But the Mw of the polysaccharides that we obtained from *R. Laevigatae* fruits were less than 10 kDa, far less than the previous studies. Previous studies have reported that the large molecular weight of polysaccharides (over 10 kDa) led to poor water solubility and unfavorable for the absorption into the body, reduced the biological activities, and possibly limited the applications of polysaccharides, whereas the low molecular weight polysaccharides less than 10 kDa had relatively higher activities to human health [18, 19].

### 3.4. Structural Analyses of RLPs

The monosaccharide compositions of RLP-1a, RLP-2a, and RLP-3a were identified by comparing the retention time with external standards by GC-MS analysis as shown in Table 3. The monosaccharide compositions showed that RLP-1a contained five different monosaccharides including ribose, arabinose, xylose, glucose, and galactose with the molar ratio of 3.14 : 8.21 : 1 : 1.37 : 4.90, whereas RLP-2a consisted of four different monosaccharides including ribose, mannose, glucose, and galactose with the molar ratio of 1.70 : 1 : 93.59 : 2.73, and RLP-3a contained six different monosaccharides including ribose, arabinose, xylose, mannose, glucose, and galactose with a molar ratio of 6.04 : 26.51 : 2.05 : 1 : 3.17 : 31.77.

**Figure 4:**
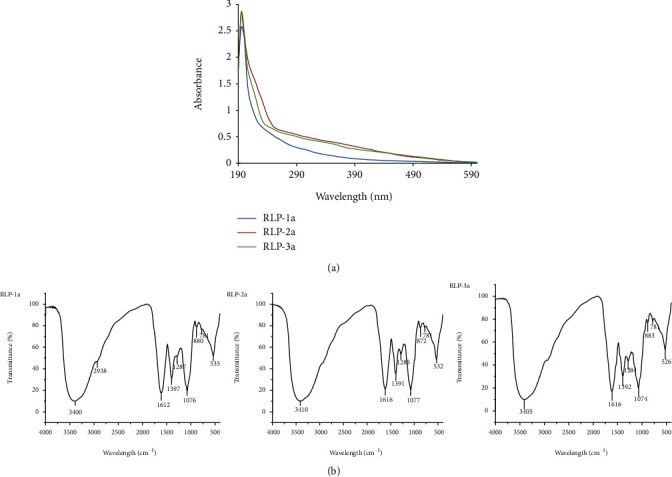
UV spectrophotometry analysis (a) and FT-IR spectrophotometry analysis (b) of the purity of low molecular weight soluble polysaccharides RLP-1a, RLP-2a, and RLP-3a from *R. Laevigatae* fruits.

RLP-1a, RLP-2a, and RLP-3a showed no absorbance peaks at 280 and 260 nm by UV scanning, revealing the absence of protein and nucleic acid in polysaccharides (Figure 4(a)). FT-IR spectroscopy is a suitable technique for the identification of characteristic organic groups in the polysaccharides. As shown in Figure 4(b), the different absorption bands of the FT-IR analysis were assigned as previously reported [25, 29]. The broad stretching peak at 3400 cm^−1^ was ascribed to the hydroxyl groups with stretching vibration. The weak absorption peaks at 2935 cm^−1^ and 2939 cm^−1^ were characteristic of C-H stretching vibration, and these bands above were characteristic absorption peaks of polysaccharides. The characteristic absorption band near 883 cm^−1^ indicated that RLP-1a, RLP-2a, and RLP-3a contained *β*-type glycosidic bond [30]. Moreover, near 1715 cm^−1^ without absorption further explained that no uronic acids existed in polysaccharides [31].

NMR spectra were employed to further confirm the obtained structure data and provide more detailed polysaccharide structural information. Generally, it is believed that there are several kinds of monosaccharides when there are several proton resonance signals in the spectrum *δ* 4.0-6.0 ppm in the ^1^H-NMR spectrum. In ^1^H-NMR, RLP-1a had six signals at *δ* 4.42, *δ* 4.56, *δ* 4.61, *δ* 5.01, *δ* 5.11, and *δ* 5.15 ppm (Table 4), indicating that there were both *α*-linkage and *β*-linkage in the glycosidic bonds of RLP-1a. There was no proton signal at *δ* 5.4 ppm, indicating that RLP-1a was composed of glucopyranose [25]. Besides, an independent methyl signal was observed at *δ*1.24 ppm. It was assigned to Rha*p* [32].

In the ^13^C-NMR spectrum of RLP-1a (Table 4), *δ* 107.46, *δ* 106.87, *δ* 102.48, *δ* 101.68, *δ* 96.06, and *δ* 91.99 ppm were shown in *δ* 90-110 ppm hetero-carbon region, indicating that there were at least six monosaccharide residues in RLP-1a. Generally, for pyranose residue attended in glycosidic linkage, the chemical shift of anomeric carbon was in the range of *δ* 100-106 ppm in *β* configuration and *δ* 93-100 ppm in *α* configuration [31]. When present as a reducing terminal, the movement to the high field was observed [32]. According to the analysis of monosaccharide composition, RLP-1a contained five monosaccharides, which were ribose, arabinose, xylose, glucose, and galactose. It suggested that *δ* 107.46 ppm signals were assigned to C-1 of the *α*-L-Araf. And the analysis of the anomeric region of the spectrum showed that the signals at *δ* 106.87, *δ* 102.48, *δ* 101.68, and *δ* 96.06 ppm were attributed to C-1 of the *α*-D-Glc*p*, *β*-D-Gal*p*, *β*-Xyl*p*, and *α*-L-Rha*p* units, respectively [33, 34]. The weak signal at *δ* 91.99 ppm might be from *α*-D-Glc*p* at the end of the chain [31]. The absence of signals from *δ* 120 to 175 ppm suggested that RLP-1a contained no hexuronic acid, acetyl amino compounds, and carboxyl [34].

Chemical shift assignments for the ^13^C-NMR and ^1^H-NMR spectra of RLP-2a were showed in Table 4. Four single peaks appeared at *δ* H 5.16, 4.24, 4.14, and 4.05 ppm. Four weak anomeric carbon signals of RLP-2a were detected, suggesting that four monosaccharides existed in each polysaccharide unit.

Chemical shift assignments for the ^13^C-NMR and ^1^H-NMR spectra of RLP-3a were showed in Table 4. In 1H-NMR spectrum, six single peaks appeared at *δ* H 5.17, 5.08, 5.01, 4.93, 4.66, and 4.43 ppm, indicating that RLP-3a contained both *α*- and *β*-anomeric configurations. Six anomeric carbon signals at *δ* C 109.16, 107.39, 106.96, 102.94, 100.82, and 99.31 ppm appeared in ^13^C-NMR spectrum, indicating that six monosaccharides existed in one unit. The signal *δ*H/*δ*C (1.15/16.45) corresponded to the -CH_3_ groups of Rha*p* residues [35].

**Figure 5:**
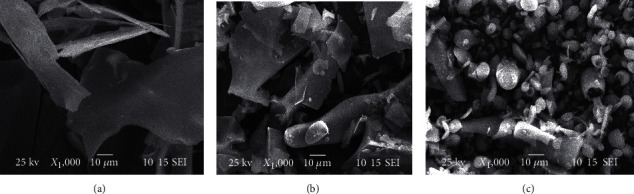
SEM images of (a) RLP-1a, (b) RLP-2a, and (c) RLP-2a.

Scanning electron microscopy (SEM) can be used to determine the surface, the internal morphology, and the pore characteristics of polysaccharides [36]. To evaluate the differences in the surface morphological structure between RLP-1a, RLP-2a, and RLP-3a, SEM was used to observe the microscopic appearances of RLP-1a, RLP-2a, and RLP-3a under 1000-fold magnification. RLP-1a had a sheet-like structure with a smooth and flat surface (Figure 5(a)), and RLP-2a was also flaky and smooth with more debris (Figure 5(b)). In contrast, the RLP-3a was present in smooth globular particles (Figure 5(c)). The microscopic morphology of the surface of the RLP-1a, RLP-2a, and RLP-3a was significantly different. The topology of the polysaccharides might be according to their different molecular arrangements [36, 37].

### 3.5. *In Vitro* Antioxidant Activity

The antioxidant activities of compounds are attributed to various reactions and mechanisms, such as radical scavenging, reductive capacity, prevention of chain initiation, and binding of transition metal ion catalysts [38]. In this work, the scavenging effects of RLP-1a, RLP-2a, and RLP-3a on hydroxyl, DPPH, and superoxide radicals were analyzed.

**Figure 6:**
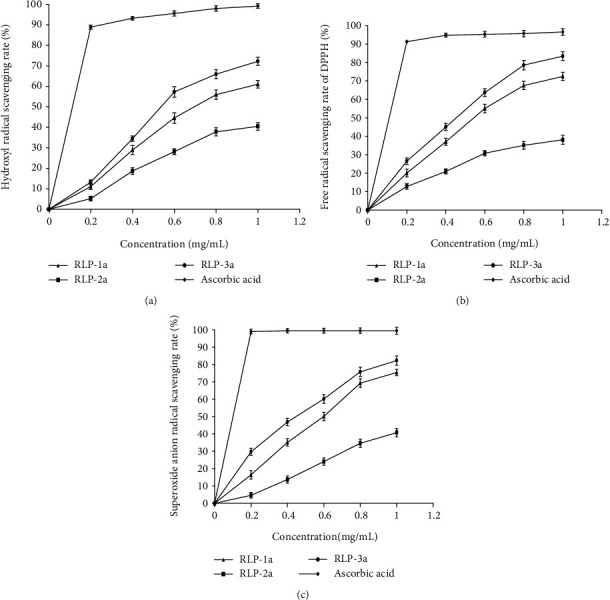
Effects of RLP-1a, RLP-2a, RLP-3a, and ascorbic acid in vitro antioxidant of (a) scavenging effects on hydroxyl radicals, (b) scavenging effects on DPPH, and (c) scavenging effects on superoxide anion radicals. The values were reported as the mean ± SD. (*n* = 3 for each group).

Hydroxyl free radical is the most active oxygen free radical, which causes various diseases including diabetes and is an important factor of oxidative damage [3]. As shown in Figure 6(a), the scavenging activity of RLP-1a, RLP-2a, and RLP-3a was concentration dependent and lower than that of ascorbic acid at the test dosage range. When the concentration was 1.0 mg/mL, the scavenging hydroxyl free radical value of RLP-3a reached 72.24 ± 1.98%, 11.21% higher than that of RLP-1a (*P* < 0.01), and 31.79% higher than that of RLP-2a (*P* < 0.01), demonstrating that RLP-3a had the highest potential scavenging effect on superoxide radicals.

DPPH, as a stable free radical, is a fast, convenient, and sensitive substance to evaluate the antioxidant activity *in vitro* [39]. As shown in Figure 6(b), the DPPH radical scavenging rates of RLP-1a, RLP-2a, and RLP-3a were concentration dependent and lower than that of ascorbic acid at the test dosage range. In the test dosage range, RLP-3a showed superior scavenging activity of DPPH radical compared with RLP-1a and RLP-2a. At 1.0 mg/mL, the scavenging rates of RLP-1a, RLP-2a, and RLP-3a reached 72.48 ± 2.19%, 38.13 ± 2.46%, and 83.46 ± 2.32%, respectively.

Superoxide anion radicals are active and have strong oxidation and toxicity. They are the first free radicals produced by oxygen in the body and easy to be converted into other active oxygen radicals, causing further oxidative damage to the body [40]. The scavenging effects of RLP-1a, RLP-2a, and RLP-3a on the superoxide radical were shown in Figure 6(c). The scavenging rates of RLP-1a, RLP-2a, RLP-3a, and ascorbic acid against superoxide radicals were directly proportional to their concentrations. In the test dosage range, the scavenging activity of ascorbic acid against superoxide radicals was remarkably higher than those of RLP-1a, RLP-2a, and RLP-3a. The scavenging superoxide rate of RLP-3a reached 82.30 ± 2.72%, and that of RLP-1a reached 75.43 ± 1.87%, which was higher than that of RLP-2a (40.79 ± 2.45%) at 1.0 mg/mL, indicating that RLP-3a and RLP-1a had superior antioxidant activities.

### 3.6. Effect of RLP on Body Weight

Changes in the body weight and food intake of rats after 12 weeks in the four groups (CN, HFD, HRLP, and LRLP) were shown in Table 5. There was no significant difference in body weight between the groups at the beginning of the experiment. However, at the end of the experiment, the weight gain of the HFD group was significantly higher than that of the CN group (*P* < 0.01). Compared with the HFD group, the HRLP and LRLP dosage groups had less weight gain (*P* < 0.01). The results indicated that RLP supplementation could inhibit the rat weight increase induced by high-fat diet.

### 3.7. Effect of RLP on Serum Lipid

**Figure 7:**
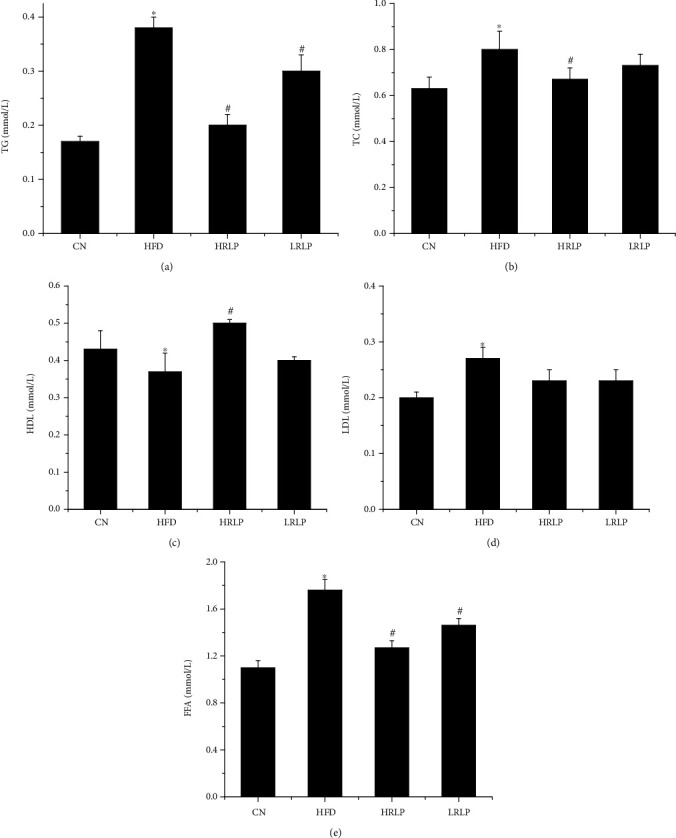
Effect of RLP on the serum lipids of HFD rats (*n* = 8, x ± s). ^∗^*P* < 0.05 vs. CN rats. ^#^*P* < 0.05 vs. HFD rats.

The effects on serum lipid were exhibited in Figure 7. Compared with the CN group, the HFD group rats displayed a significant increase of TC, TG, LDL-C, and FFA levels, but the HDL-C levels significantly decreased (*P* < 0.05). With RLP intervention, LRLP and HRLP showed hypolipidemic activities. Compared with the HFD group, the HRLP group reduced TG (*P* < 0.05), TC (*P* < 0.05), FFA (*P* < 0.05), and LDL-C (*P* < 0.1) levels by 16.3%, 47.0%, 27.8%, and 14.8%, respectively, and the HDL-C levels significantly increased by 35.0% (*P* < 0.05), indicating that RLP with higher dosage intervention had stronger hypolipidemic activities in rats, and the hypolipidemic activity of LRLP was weaker than that of HRLP.

High-fat diet induction reduced HDL-C levels and significantly increased TC, TG, and LDL levels, leading to lipid metabolism disorders. This might be due to the increased FFA accumulation in serum [41]. FFA could increase liver uptake resulting in excessive lipid accumulation, which was poisonous to liver and eventually caused hepatic steatosis [42]. After treatment with RLP, these levels of TC, TG, FFA, HDL-C, and LDL-C were distinctly relieved compared with HFD. Compared with the polysaccharides with high molecular, RLP showed more positive effects on reducing serum lipid levels in the high-dose group [1].

### 3.8. Antioxidant Status in the Liver

**Figure 8:**
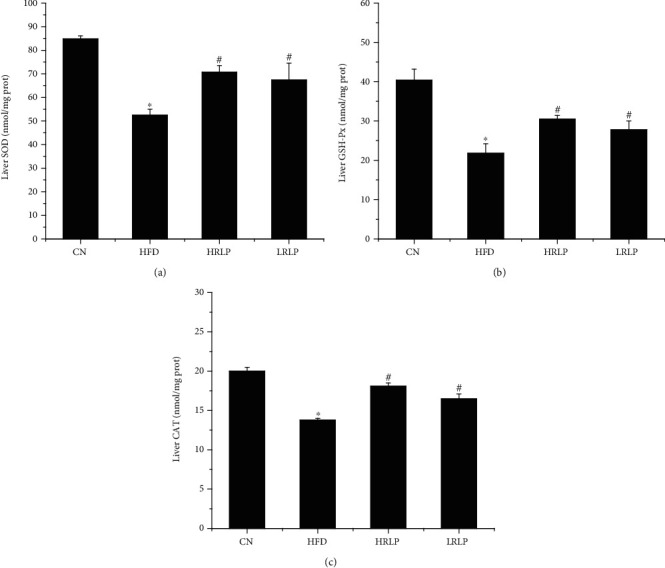
Effects of RLP on the activity of SOD, GSH-Px, and CAT in the liver of high-fat diet-fed rats (*n* = 8, x ± s). ^∗^*P* < 0.01 vs. CN rats. ^#^*P* < 0.01 vs. HFD rats.

The effects of RLP on SOD, GSH-Px, and CAT activities in the livers of rats in different experimental groups were assessed in this study. As shown in Figure 8, the activities of SOD, GSH-Px, and CAT in the HFD group livers were significantly lower than those in the CN group (*P* < 0.01). Hyperlipidemia could reduce the activity of the antioxidant enzyme system, leading to peroxidation damage [43], whereas excessive free radicals could cause lipid peroxidation, further destroyed the lipid metabolism pathway in the liver and aggravated hyperlipidemia [44]. Compared with the HFD group, the SOD, GSH-Px, and CAT activity significantly increased in livers in the LRLP group with 28.4%, 27.5%, and 19.6% (*P* < 0.01) and in the HRLP group with 34.7%, 39.9%, and 31.2% (*P* < 0.01), indicating that HRLP had a significant effect on improving the antioxidant capacity of liver tissue. These results indicated that RLP could regulate the antioxidant enzyme system to resist the oxidative stress damage caused by the high-fat diet.

### 3.9. Functional Analysis of DEGs

**Figure 9:**
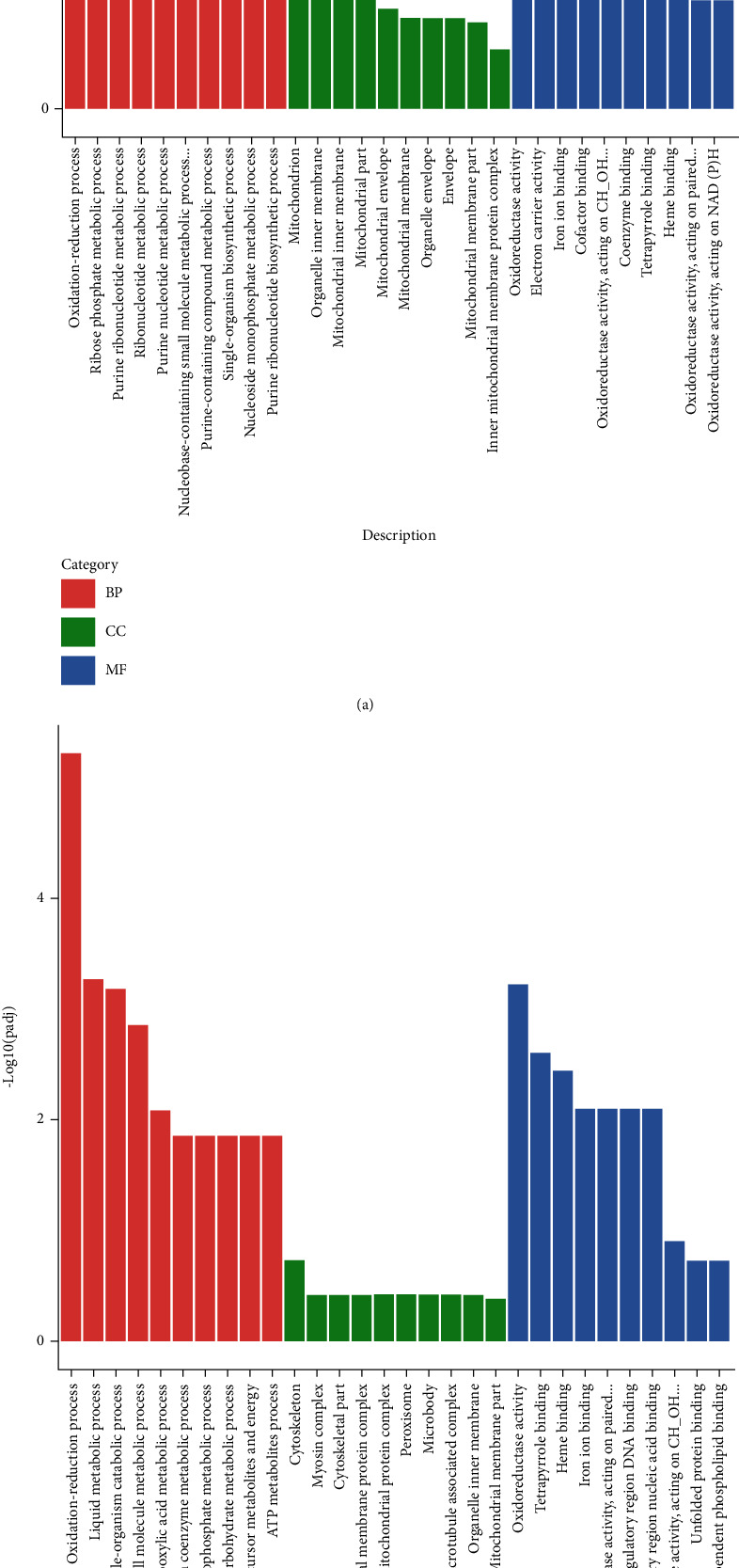
Effects of HFD and HRLP on related GO classification: (a) GO classification of DEGs between HFD and CN groups; (b) GO classification of DEGs between HRLP and HFD groups.

GO is mainly to solve the confusion of the definition of the same genes in different databases and the confusion of the same genes in different species in the functional definition. GO has three ontologies, which describe the biological process (BP), cellular component (CC), and molecular function (MF) of genes. DEGs between HFD and CN groups were assigned to 391 biological processes, 103 cellular components, and 260 molecular functions. For DEGs between HRLP and HFD, 670 GO terms were significantly enriched, including 321 biological processes, 105 cellular components, and 244 molecular functions. For biological processes, the oxidation-reduction process and lipid metabolic process were differently regulated between the two groups (Figures 9(a) and 9(b)). Oxidation-reduction process and lipid metabolic process were mainly upregulated between HRLP and HFD and downregulated in the HFD vs. CN group.

### 3.10. KEGG Pathway Analysis of DEGs

**Figure 10:**
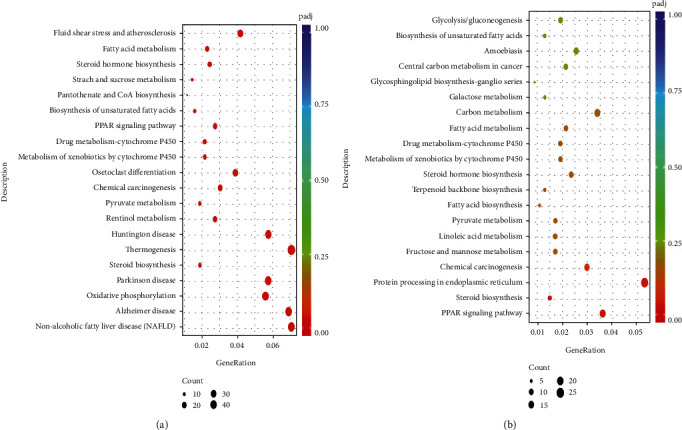
Effects of HFD and HRLP on related pathways: (a) KEGG analysis of DEGs between HFD and CN groups; (b) KEGG analysis of DEGs between HRLP and HFD groups.

Compared to the CN group, 305 pathways were obtained with HFD treatment. In contrast, compared with the HFD group, 290 important pathways were acquired with HRLP treatment, respectively, and the top 20 pathways were illustrated in (Figures 10(a) and 10(b)). Both HFD and HRLP regulated four pathways including peroxisome proliferator-activated receptor (PPAR) signaling pathway, fatty acid metabolism, steroid biosynthesis, and linoleic acid metabolism. We selected the PPAR signaling pathway to evaluate the mechanism by RLP-regulated HFD-induced metabolic dysfunction. For genes in the PPAR signaling pathway, compared with CN group, the expression of acyl coenzyme A oxidase 3 (*ACOX3*) (*P* < 0.05), which limited the fatty acid *β*-oxidation pathway significantly, reduced in HFD group, and the expression of *ACOX3* in HRLP group was significantly higher than that in HFD group, indicating that RLP intervention could effectively reverse the downregulation of *ACOX3* caused by the high-fat diet. Fatty acyl-CoA imported to the mitochondria was desaturated to 2-trans-enoyl-CoAs by *ACOX3* and further processed into *β*-oxidation [45]. Our results suggested that *ACOX3* expression was upregulated under RLP feeding conditions, promoted the decomposition of fatty acids, and reduced fat accumulation in the liver. Moreover, two significant down expressed key genes of lipid synthesis fatty acid desaturase 2 (*FADS2*) and stearoyl-coenzyme A desaturase 1 (*SCD-1*) (*P* < 0.05) were found between group HFD vs. CN, which played a central role in the metabolism of long-chain fatty acids. Under the intervention of RLP, the expressions of these genes were reversed.

*FADS2* is a lipid biosynthetic protein. Linoleic acid is continuously converted into arachidonic acid in a pathway regulated by the *FADS2* gene. The fatty acid dehydrogenase synthesized by *FADS2* is a key enzyme in the biosynthesis of unsaturated fatty acids, which is highly expressed in the liver. It has been reported that *FADS2* has an inhibitory effect on hepatic lipid accumulation [46]. Our results revealed that *FADS2* expression was the highest under RLP feeding conditions. *SCD-1* plays an important role in both hepatocyte apoptosis mediation and fatty acid metabolism. *SCD-1* is an endoplasmic reticulum enzyme, which catalyzes the rate-limiting step in monounsaturated fatty acid formation and is required to guard against dietary unsaturated fat deficiency [47]. Despite the abundance of monounsaturated fatty acids (MUFAs) in dietary fats, MUFAs are also synthesized *de novo* from saturated fatty acids by *SCD-1* enzymes. *SCD-1* deficient rats experience reduced MUFA synthesis, decreased HDL cholesterol levels, decreased lipid accumulation, and enhanced fatty acid oxidation [48–50]. Our data showed that *SCD-1* was downexpressed by HFD feeding and increased by RLP intervention, indicating that nonalcoholic fatty liver caused by HFD led to decrease in *SCD-1*. After taking RLP, the expression level of *SCD-1* increased, resulting in a large increase in HDL cholesterol and a lipid accumulation decrease in the liver of HFD rats.

**Figure 11:**
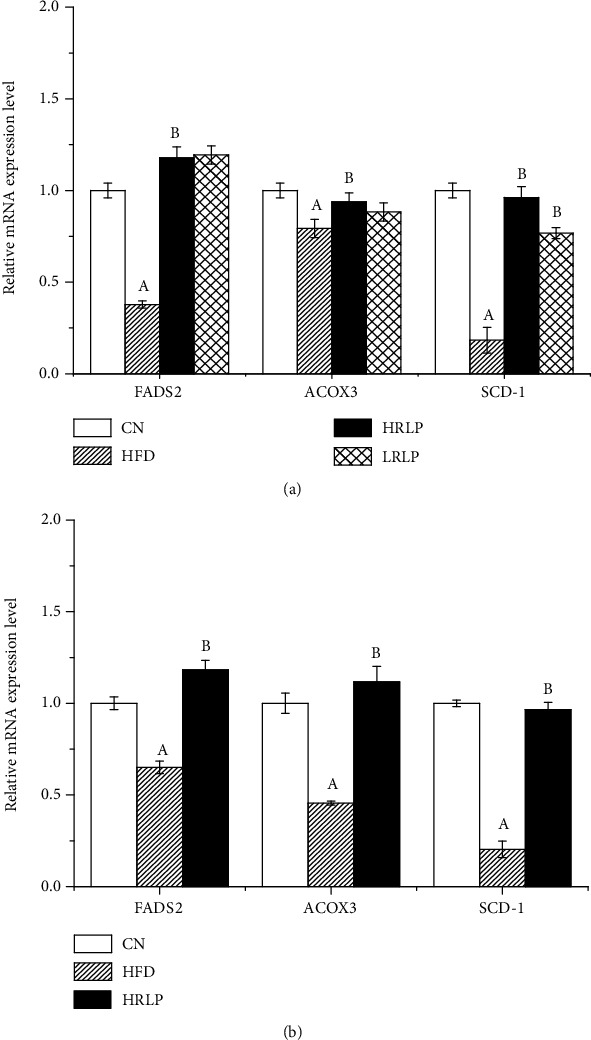
Effects of HFD and HRLP on liver gene expression of *FADS2*, *ACOX3*, and *SCD-1*. (a) mRNA expressions were determined by RNA-Seq, and set the fold change of the control group to 1 and then calculate the fold change for each group (*n* = 3). (b) mRNA expressions were determined by qRT-PCR, and set the fold change of the control group to 1 and then calculate the fold change for each group (*n* = 3); (a) significantly different from the corresponding normal chow (CN) group (*P* < 0.05); (b) significantly different from the corresponding high-fat diet (HFD) group (*P* < 0.05).

mRNA expressions of *ACOX3*, *FADS2*, and *SCD-1* were genes involved in fatty acid metabolism and play an important role in the PPAR signaling pathway. It was noted that RLP treatment at 800 mg/kg doses significantly promoted the expression of peroxisome proliferator-activated receptor alpha (*PPARa*) target genes *ACOX3* and increased the expression of lipid synthesis genes such as *FADS2* and *SCD-1* expression (*P* < 0.05). The RNA-Seq investigation was consistent with the results of the qRT-PCR analyses (Figures 11(a) and 11(b)).

## 4. Conclusions

Three novel low molecular weight polysaccharides RLP-1a, RLP-2a, and RLP-3a of 9004 Da, 8761 Da, and 7571 Da, respectively, were purified from the *R. Laevigatae* fruits, which had 6, 4, and 6 types of glycosidic linkages, respectively. The present study demonstrated that these RLPs exhibited the distinct hypolipidemic and antioxidant effects in high-fat diet-induced rats. RLPs enhanced antioxidant enzyme activities, protected liver cells, improved serum lipid profiles, and inhibited lipid accumulation in the liver by upregulation of *FADS2*, *ACOX3*, and *SCD-1* involved in the lipid metabolic processes and the oxidative stress. RLPs with low molecular weight attenuated the HFD-induced dysfunction of fat metabolic and liver functions, as well as oxidative processes. The fat metabolic dysfunction was ameliorated by RLPs through the integration of the PPAR signaling pathway, suggesting a new route for possible treatment of hyperlipidemia. RLPs had potential applications as a candidate in the functional food supplements for hyperlipidemia population.

## Figures and Tables

**Table 1:** Response surface central composite design and results for extraction yield of RLP.

Std. order	*X*_1_, extraction temperature (°C)	*X*_2_, extraction time (h)	*X*_3_, water to raw material ration (mL/g)	*X*_4_, extraction frequency	Extraction yield of RLP (%)
1	-1(85)	-1(1.5)	-1(1 : 18)	-1(2)	7.77 ± 0.33
2	-1(85)	-1(1.5)	-1(1 : 18)	1(4)	8.97 ± 0.39
3	-1(85)	-1(1.5)	1(1 : 22)	-1(2)	7.91 ± 0.14
4	-1(85)	-1(1.5)	1(1 : 22)	1(4)	7.19 ± 0.35
5	-1(85)	1(2.5)	-1(1 : 18)	-1(2)	8.38 ± 0.25
6	-1(85)	1(2.5)	-1(1 : 18)	1(4)	8.27 ± 0.03
7	-1(85)	1(2.5)	1(1 : 22)	-1(2)	8.41 ± 0.17
8	-1(85)	1(2.5)	1(1 : 22)	1(4)	8.26 ± 0.15
9	1(95)	-1(1.5)	-1(1 : 18)	-1(2)	7.71 ± 0.02
10	1(95)	-1(1.5)	-1(1 : 18)	1(4)	8.97 ± 0.37
11	1(95)	-1(1.5)	1(1 : 22)	-1(2)	8.2 ± 0.04
12	1(95)	-1(1.5)	1(1 : 22)	1(4)	8 ± 0.02
13	1(95)	1(2.5)	-1(1 : 18)	-1(2)	7.62 ± 0.2
14	1(95)	1(2.5)	-1(1 : 18)	1(4)	8.01 ± 0.12
15	1(95)	1(2.5)	1(1 : 22)	-1(2)	9.49 ± 0.1
16	1(95)	1(2.5)	1(1 : 22)	1(4)	8.6 ± 0.21
17	-2(80)	0(2)	0(1 : 20)	0(3)	8.09 ± 0.44
18	2(100)	0(2)	0(1 : 20)	0(3)	8.63 ± 0.02
19	0(90)	-2(1)	0(1 : 20)	0(3)	8.38 ± 0.03
20	0(90)	2(3)	0(1 : 20)	0(3)	9.24 ± 0.36
21	0(90)	0(2)	-2(1 : 16)	0(3)	7.53 ± 0.31
22	0(90)	0(2)	2(1 : 24)	0(3)	7.7 ± 0.36
23	0(90)	0(2)	0(1 : 20)	-2(1)	7.22 ± 0.03
24	0(90)	0(2)	0(1 : 20)	2(5)	7.78 ± 0.03
25	0(90)	0(2)	0(1 : 20)	0(3)	9.36 ± 0.07
26	0(90)	0(2)	0(1 : 20)	0(3)	9.26 ± 0.06
27	0(90)	0(2)	0(1 : 20)	0(3)	9.39 ± 0.37
28	0(90)	0(2)	0(1 : 20)	0(3)	9.29 ± 0.04
29	0(90)	0(2)	0(1 : 20)	0(3)	9.22 ± 0.03
30	0(90)	0(2)	0(1 : 20)	0(3)	8.7 ± 0.16

**Table 2:** ANOVA for response surface quadratic model.

Variables	Sun of squares	DF	Mean square	*F* value	*P* value
*X*_1_	0.2646	1	0.2646	3.3678	0.0864^b^
*X*_2_	0.6801	1	0.6800	8.6558	0.0101^a^
*X*_3_	0.0204	1	0.0204	0.2599	0.6176^b^
*X*_4_	0.1504	1	0.1504	1.9145	0.1867^b^
*X*_1_ *X*_2_	0.0256	1	0.0256	0.3258	0.5766^b^
*X*_1_ *X*_3_	0.81	1	0.81	10.3096	0.0058^a^
*X*_1_ *X*_4_	0.007225	1	0.0072	0.09196	0.7659^b^
*X*_2_ *X*_3_	1.3225	1	1.3225	16.8326	0.0009^a^
*X*_2_ *X*_4_	0.3306	1	0.3306	4.2082	0.0581^b^
*X*_3_ *X*_4_	1.3806	1	1.3806	17.5724	0.0008^a^
*X*_1_^2^	0.9240	1	0.9240	11.7606	0.0037^a^
*X*_2_^2^	0.1384	1	0.1384	1.7619	0.2042^b^
*X*_3_^2^	3.7507	1	3.7507	47.7390	<0.0001^a^
*X*_4_^2^	4.3566	1	4.3566	55.4506	<0.0001^a^
Model	12.3418	14	0.8816	11.2204	<0.0001^a^
Residual	1.1785	15	0.0786		
Lack of fit	0.8548	10	0.0855	1.3202	0.4001
Pure error	0.3237	5	0.0647		
Cor total	13.5204	29			
*R* ^2^ = 0.9128					
*R*_adj_^2^ = 0.8315					
C.V.% = 3.3429					

^a^Significance (*P* < 0.05); ^b^ nonsignificance (*P* > 0.05).

**Table 3:** Monosaccharide composition of low molecular weight soluble polysaccharides RLP-1a, RLP-2a, and RLP-3a from *R. Laevigatae* fruits.

Polysaccharides fractions	Monosaccharide composition (molecular ratio (%))					
	Rha	Ara	Xyl	Man	Glc	Gal
RLP-1a	3.14 ± 0.20	8.21 ± 0.09	1.0 ± 0.02	—	1.37 ± 0.44	4.9 ± 0.13
RLP-2a	1.70 ± 0.05	—	—	1.0 ± 0.04	93.59 ± 0.1	2.73 ± 0.10
RLP-3a	6.04 ± 0.06	26.51 ± 0.08	2.05 ± 0.03	1.0 ± 0.02	3.17 ± 0.07	31.77 ± 0.09

**Table 4:** Chemical shifts of resonances in the ^1^H and ^13^C spectra of RLP-1a, RLP-2a, and RLP-3a (ppm).

Polysaccharide fractions	Residues	C1/H1	C2/H2	C3/H3	C4/H4	C5/H5	C6/H6
RLP-1a	(1→6)-*α*-D-Glc*p*	106.87/5.11	76.64/3.88	75.91/3.16	73.27/3.16	72.82/3.73	69.56/3.31
	(1→4)-*α*-L-Araf*p*	107.46/5.01	76.52/3.39	77.76/3.73	81.34/4.06	75.91/3.32	69.56/4.06
	(1→)-*α*-D-Glc*p* terminal	91.99/5.15	76.64/3.16	76.20/4.06	75.76/3.31	69.50/3.31	60.75/3.73
	(1→3,4)-*β*-D-Xyl*p*	101.68/4.61	75.76/3.39	82.03/3.73	71.42/3.16	60.75/3.64	-/-
	(1→6)-*β*-D-Gal*p*	102.48/4.42	69.57/3.31	72.72/3.73	71.42/3.80	60.75/4.08	69.15/3.31
	(1→3)-*β*-L-Rha	96.06/4.56	79.73/4.21	81.34/4.06	75.76/3.32	69.56/4.06	60.75/3.31
RLP-2a	(1→4)-*α*-D-Glc*p*	109.30/5.16	75.95/3.96	75.81/3.31	89.37/3.96	70.09/3.45	60.92/3.74
	(1→5)-*β*-D-Man*p*	104.55/4.14	75.91/3.38	89.37/3.45	75.81/3.69	81.43/4.15	61.12/3.96
	(1→4)-*β*-D-Gal*p*	103.43/4.24	76.64/3.88	76.20/4.06	81.43/4.14	75.81/3.41	61.12/3.64
	(1 → 2)-*β*-L-Rha*p*	100.52/4.05	81.43/4.14	75.95/3.85	70.09/3.74	68.58/3.77	60.92/3.77
RLP-3a	(1→4,6)-*α*-L-Araf*p*	109.16/5.17	75.71/3.42	72.27/3.42	81.66/4.06	73.28/3.4	69.51/3.62
	(1→5)-*α*-D-Glc*p*	107.39/5.01	72.27/3.93	75.71/3.42	68.78/3.93	81.20/4.06	60.97/3.75
	(1→6)-*α*-D-Gal*p*	106.96/5.08	66.09/3.81	75.71/4.06	83.36/3.86	61.09/3.65	68.78/3.93
	(1→6)-*β*-D-Man*p*	102.94/4.43	68.78/3.86	81.20/4.14	61.09/3.75	60.97/3.60	66.09/3.81
	(1→4)-*β*-D-Xyl*p*	100.82/4.66	72.27/3.60	68.78/3.93	83.36/3.93	60.75/4.08	-/-
	(1→2)-*β*-L-Rha*p*	99.31/4.93	81.66/4.14	69.52/3.31	75.71/3.42	66.09/3.81	61.09/3.65

**Table 5:** Effects of RLP on body weight of different groups of rats.

	Body weight (g)		
	Initial	Final	Growth rate (%)
CN	340.63 ± 8.2	503.66 ± 10.3	48.14
HFD	337.75.3 ± 7.4	654.66 ± 11.2^∗∗^	93.80
HRLP	352.38 ± 6.0	601.00 ± 4.6^##^	70.55
LRLP	350.50 ± 7.1	616.76 ± 7.6^##^	76.00

^∗∗^ *P* < 0.01: compared with the CN group. ^#^*P* < 0.05: compared with HFD group. ^##^*P* < 0.01: compared with HFD group.

## Data Availability

The data used to support the findings of this study are available from the corresponding author upon request.
